# Enhanced optical force on multilayered dielectric nanoparticles by tuning material properties and nature of excitation: a theoretical investigation

**DOI:** 10.1039/d2na00280a

**Published:** 2022-06-06

**Authors:** Sumit Yadav, Anita Devi, Arijit K. De

**Affiliations:** Condensed Phase Dynamics Group, Department of Physical Sciences, Indian Institute of Science Education and Research (IISER) Mohali Knowledge City, Sector 81 SAS Nagar Punjab 140306 India; Condensed Phase Dynamics Group, Department of Chemical Sciences, Indian Institute of Science Education and Research (IISER) Mohali Knowledge City, Sector 81 SAS Nagar Punjab 140306 India akde@iisermohali.ac.in

## Abstract

Using dipole approximation, a comparative study of trapping force/potential on different types of dielectric nanoparticles is presented. The trapping force for multilayered nanoparticles, *i.e.* core–shell–shell type nanoparticles, is found to be enhanced compared with both core-only type and core–shell type nanoparticles. It is shown that an appropriate choice of material and thickness of the middle layer results in tuning the polarizability, thereby playing a vital role in determining the trapping efficiency for core–shell–shell type nanoparticles. Further, the effect of optical nonlinearity under femtosecond pulsed excitation is investigated and it is elucidated that depending on the specific need (*i.e*. high force *versus* long confinement time), the nature of excitation (*i.e.* pulsed excitation or continuous-wave excitation) can be judiciously chosen. These findings are promised to open up new prospects for controlled nanoscale trapping and manipulation across different fields of nanoscience and nanotechnology.

## Abstract 2

Using dipole approximation, a comparative study of trapping force/potential on bare, core/shell and core/shell/shell types of dielectric nanoparticles is presented. The optimal conditions for high-force or long confinement time are discussed.

## Graphical abstract

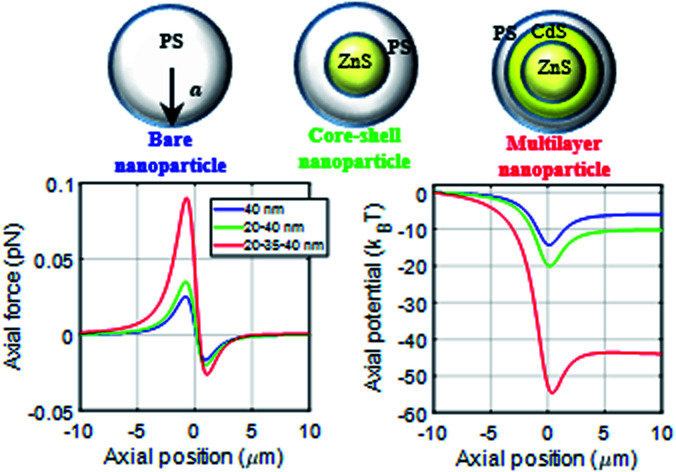

## Introduction

Facile manipulation of nanoparticles can be performed using optical trapping^1^ to result in wide-ranging applications in nanoscience and nanotechnology.^2–5^ Optical trapping of dielectric nanoparticles still remained as a challenging task due to the erratic Brownian motion of these particles which increases with decreasing dimensionality. Consequently, to get a stable trap for such particles, very high laser power is used which is otherwise detrimental considering the laser-induced heating, *etc.* effects. As an alternative, *repetitive instantaneous trapping* through the use of high repetition-rate ultrafast pulsed excitation was envisaged.^6^ Subsequently, it was realized that under such excitation, nonlinear phenomena, such as the optical Kerr effect (OKE), must be taken into account and OKE was shown to dramatically modulate the trapping efficiency.^7^ It was demonstrated that the trapping efficiency is characterized by the height of potential barrier to escape the trapping well, *i.e. escape potential*.^7^ Further studies revealed the usefulness of exploring as well as harnessing optical nonlinearity under pulsed excitation for dielectric^8–10^ nanoparticles. Of special interest is to tailor the polarizability of hybrid (*i.e.* composed of different materials in layered structures) nanoparticles due to their wide-ranging interesting applications, for example, electromagnetic cloaking using multilayered nanoparticles^11^ or enhancing spatial resolution without any compromise with the size of particles during constant force measurement in bioconjugated experiment.^12,13^ In recent work, we theoretically demonstrated enhanced trapping efficiency for core–shell type nanoparticles over bare ones which was further shown to be fine-tuned by optical nonlinearity under ultrashort pulsed excitation.^14^ Also, a reversal (from repulsive to attractive) in optical trapping force under pulsed excitation was reported.^14–16^ However, due to the facile tuneability of effective polarizability by changing the thickness of layers of multilayered nanoparticles are promised to offer better trapping efficiency.

In this article, we present a comparative study of force and potential on core-only or bare type (polystyrene), core–shell (ZnS–polystyrene) type, and core–shell–shell (ZnS–CdS–polystyrene) type nanoparticles using dipole approximation under both continuous-wave (CW) and pulsed excitation. A significant enhancement in trapping efficiency is observed for core–shell–shell type nanoparticles as compared to bare and core–shell type nanoparticles. It is shown that an appropriate choice of material composition and thickness of the middle layer, *i.e.* inner shell, can significantly enhance trapping efficiency for the core–shell–shell type nanoparticles, resulting in stable trapping of nanoparticles. Further, we show the effect of optical nonlinearity under pulsed excitation, which can dramatically influence the trapping efficiency of these nanoparticles. Most importantly, the present work shows how a judicious choice of nature of excitation, CW or pulsed excitation, can have certain advantage depending on the specific application, whether an impulsive force is to be exerted on the particles or they need to be trapped for longer time.

## Mathematical formulation

Considering the wide use of polystyrene particles in bio-conjugate experiments,^17,18^ we choose different types of layered nanoparticles having outer layer as polystyrene. We compare the results for ZnS–polystyrene as core–shell nanoparticle, and ZnS–CdS–polystyrene as core–shell–shell nanoparticle with that of core-only type polystyrene nanoparticles. Using a tightly focused Gaussian beam, and corresponding intensity can be written as:^19^1
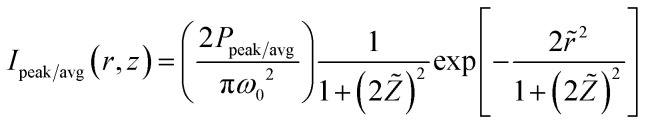
where, 
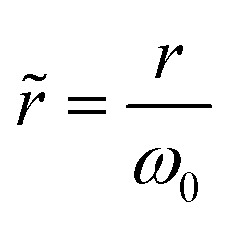
 and 
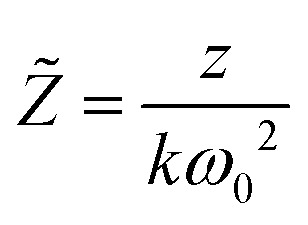
 are the reduced coordinates; 
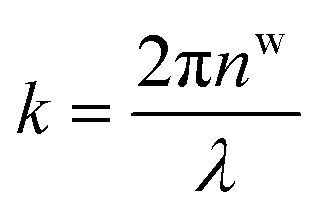
 is wavenumber inside the medium, *n*^w^ is the refractive index (RI) of the surrounding medium (water), 
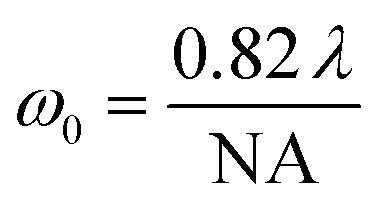
 is spot-size at focus, *λ* is the wavelength of trapping beam, NA is the numerical aperture of the objective, and *P*_peak/avg_ is average power under CW and peak power under pulsed excitation; here 
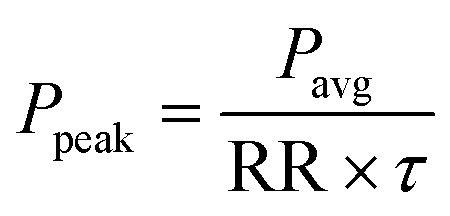
; RR and *τ* are the repetition rate and pulse-width, respectively. In the paraxial approximation of zeroth-order Gaussian beam, along the axial direction, only gradient force exists^19^ and the trap is always stable due to the restoring nature of this force. Note in the tight focusing condition like for high NA (1.4), the above approximation is not exactly applicable for accurate quantitative results. Here we used it for the qualitative comparison of force and potential. For an exact calculation of force/potential more rigorous theories, like the Generalized Lorenz-Mie theory or exact Mie theory or Maxwell stress tensor theory, can be used. However, along the axial direction, gradient and scattering forces act on the particles, and for stable trapping a delicate balance between both the forces is needed. Therefore, the trapping force and potential are calculated along the axial direction only; mathematical expressions for these forces can be written as:^19^2
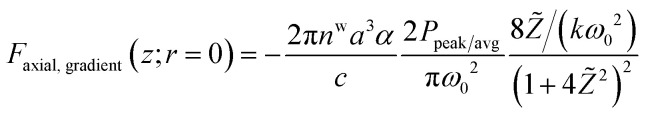
3
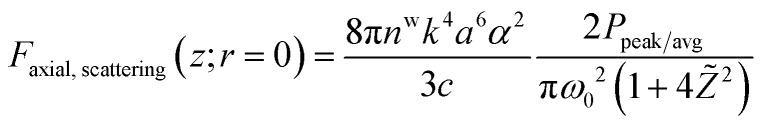


Here, *c* is the speed of light, *a* is the overall radius of the particle, and *α* is the polarizability per unit volume. The polarizability is a major factor in determining the magnitude as well as the direction of force experienced by the particle. For the bare nanoparticle, the *α* can be written as:4a
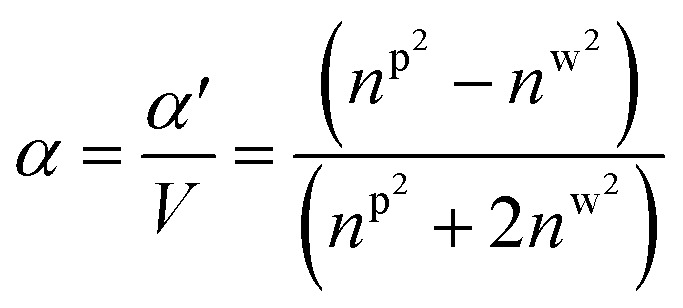


Here, *n*^p^ is the RI of the particle and *V* is 4π*n*^w^2^^*ε*_0_*a*^3^. For the core–shell type nanoparticles, *α* can be calculated as:^9,20^4b
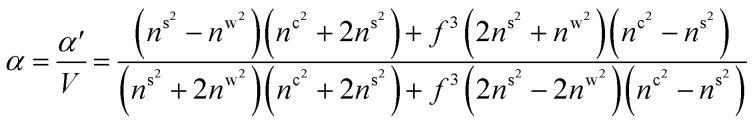
where, *n*^c^, and *n*^s^ are the RI of core, and shell, respectively. 
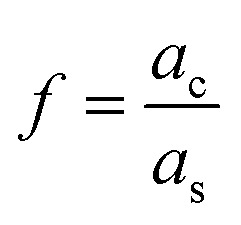
 is the ratio of core radius (*a*_c_) to that of shell (*a*_s_), and *V* is 4π*n*^w^2^^*ε*_0_*a*^3^_s_. For the core–shell–shell type nanoparticles, *α* is calculated by considering individual polarizability factors of the core, the inner shell, and shell2 which can be expressed as:^20,21^4c
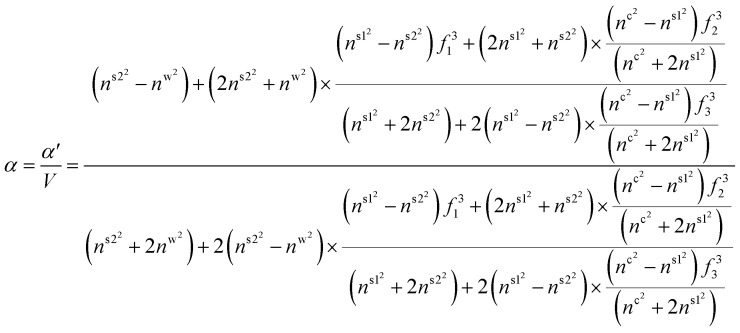
where, *n*^c^, *n*^s1^, and *n*^s2^ are the RI of core, the inner shell, and shell2, respectively. The 
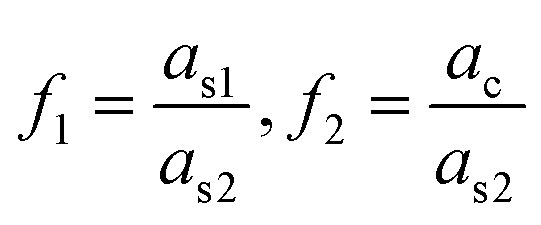
, and 
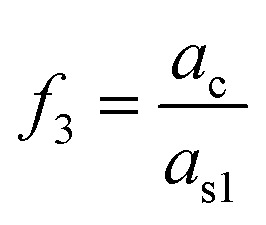
 are the ratio of the radius of the inner shell (*a*_s1_) to that of shell2 (*a*_s2_), the ratio of the radius of core (*a*_c_) to that of shell2 (*a*_s2_), and the ratio of the radius of core (*a*_c_) to that of the inner shell (*a*_s1_), respectively, and *V* is 4π*n*^w^2^^*ε*_0_*a*^3^_s2_. If *n*^c^ = *n*^s1^ = *n*^s2^ = *n*^p^, we get the *α* equivalent to core-type nanoparticle, as mentioned in eqn (4a).

Further, nonlinear effects (due to optical Kerr effect, OKE) are taken into account in a phenomenological way:^22^*n*^w/c/s1/s2^ = *n*^w/c/s1/s2^_0_ + *n*^w/c/s1/s2^_2_ × *I*_avg/peak_(*r*, *z*). Since the NRI for water is very low (refer to Table 1), it does not contribute much to the total RI at low average power (<1 W) and can be neglected for both CW and pulsed excitation. For particle RI, nonlinearity does not contribute significantly under CW excitation; however, under pulsed excitation, nonlinear RI contributes significantly to the linear RI of the particle. The refractive index including optical nonlinearity can be expressed as:*n*^p^ = *n*^p^_0_ + *n*^p^_2_ × *I*

**Table 1:** List of the parameters used in numerical calculations. NRI: nonlinear refractive index

Parameters used	Symbol	Value/expression
Central wavelength	*λ*	800 nm
Speed of light	*c*	3 × 10^8^ m s^−1^
Repetition rate	*f*	76 MHz
Pulse width	*τ*	120 fs
Numerical aperture	NA	1.4
Linear RI of the medium^23^	*n* ^w^_0_	1.329
2^nd^ order NRI of water^7^	*n* ^w^_2_	2.7 × 10^−20^ m^2^ W^−1^
Linear RI of polystyrene^23^	*n* ^s2^_0_	1.578
2^nd^ order NRI of polystyrene^24^	*n* ^s2^_2_	5.9 × 10^−17^ m^2^ W^−1^
Linear RI of CdS^23^	*n* ^s1^_0_	2.386
2^nd^ order NRI of CdS^25^	*n* ^s1^_2_	1.68 × 10^−18^ m^2^ W^−1^
Linear RI of ZnS^23^	*n* ^c^_0_	2.313
2^nd^ order NRI of ZnS^25^	*n* ^c^_2_	3.8 × 10^−19^ m^2^ W^−1^

Under CW excitation, at 100 mW average power, the nonlinear part is ignored because its contribution is significantly less, for example: For polystyrene: *n*^p^_0_ = 1.578, *n*^p^_2_ = 5.9 × 10^−17^, *I* ≈ 2.23 × 10^11^, nonlinear term (*n*^p^_2_ × *I*) is ∼1.3 × 10^−5^, so this can be ignored. Under pulsed excitation: *I*_peak_ = 2.45 × 10^16^, peak power of ∼11 kW corresponding to the average power 100 mW, and the nonlinear term (*n*^p^_2_ × *I*) is ∼1.45, which cannot be ignored. Thus, in our calculation, we consider (under CW excitation):

5a *n*^w/c/s1/s2^ ≈ *n*^w/c/s1/s2^_0_

Under pulsed excitation:*n*^w^ ≈ *n*^w^_0_

5b *n*^c/s1/s2^ = *n*^c/s1/s2^_0_ + *n*^c/s1/s2^_2_ × *I*_peak_(*r*, *z*)

Here, notation in the superscript represents core (c), inner shell (s1), and shell2 (s2). The essential parameters used in the calculations are listed in Table 1. Note, here for the used parameters under pulsed excitation, peak intensity is ∼1.2 × 10^11^ W cm^−2^ corresponding to the 100 mW average power with 120 fs pulse duration and 76 MHz repetition rate at the focal spot (*ω*_0_ = 500 nm), which is much less than the threshold of dielectric breakdown (10^14^ W cm^−2^),^26^ so the particles can be trapped at this power level without damage. The total force (gradient + scattering) is calculated along the axial direction using eqn (2) and (3). Under CW excitation, the force experienced by the particle is equivalent to the average force:

6a *F*_CW_ ≡ *F*_avg_

In case of pulsed excitation, the total force is calculated by time averaging over one duty cycle, which can be written as:6b
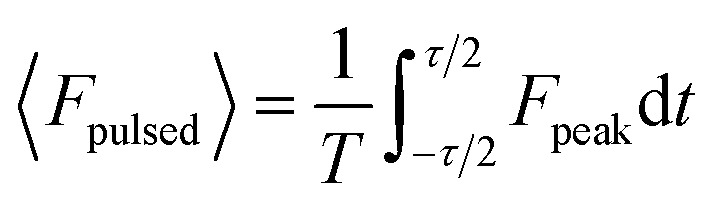


The potential is calculated by numerically integrating the force expressions:7a
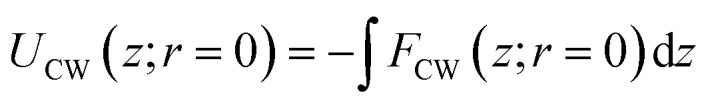
7b
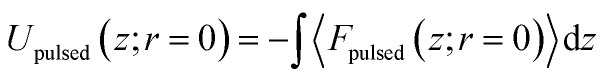


## Results and discussion

We numerically simulate the force and potential on hybrid nanoparticles keeping the overall radius of particle fixed as 40 nm and average power as 100 mW, unless mentioned otherwise. As discussed earlier the outer layer is chosen as polystyrene keeping in mind of the compatibility of bio-conjugate experiments as well as feasibility of synthesis of these nanoparticles^27^ and different combinations of layer thickness are studied to get the optimal values of parameters for both high trapping force and long trapping stability.

**Fig. 1:**
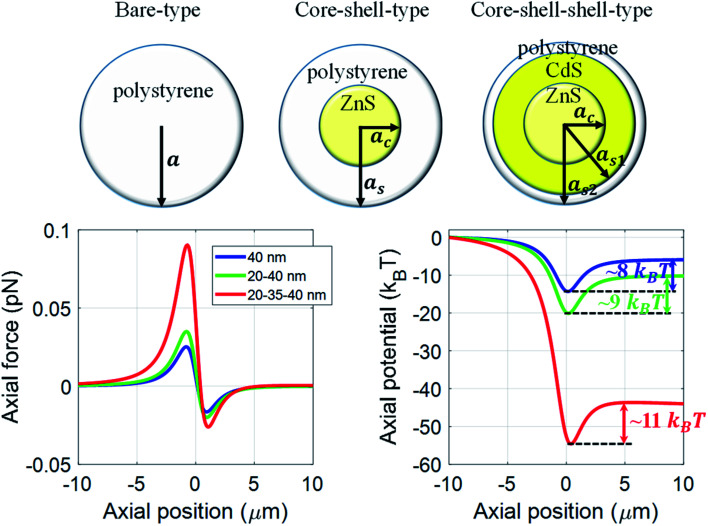
Trapping force and potential for polystyrene/ZnS–polystyrene/ZnS–CdS–polystyrene nanoparticles under CW excitation at 100 mW average power. Color: blue/green/red curve represents the polystyrene/ZnS–polystyrene/ZnS–CdS–polystyrene nanoparticles, respectively.

Fig. 1 shows trapping force and potential for the bare, core–shell, and core–shell–shell type nanoparticles under CW excitation. A 20–40 nm ZnS–polystyrene nanoparticle shows a significant enhancement in the force as compared to 40 nm polystyrene nanoparticle because in ZnS–polystyrene nanoparticle, the RI of the core is greater than that of the shell which results a change in the effective polarizability of the particle. A further enhancement in the force can be evident if a layer of different material is considered in between the ZnS–polystyrene nanoparticle making it three-layered nanoparticles. As shown in Fig. 1, if a 15 nm of CdS layer is considered between the ZnS–polystyrene nanoparticle, approximately 2.5 times enhancement in the force maxima is observed as compared to ZnS–polystyrene nanoparticle. This is because the RI of the core and the inner shell is higher than the outer shell, resulting in a significant enhancement in the overall polarizability and consequently enhances the force and the potential. This enhancement in the force is a clear indication of the advantage of using core–shell–shell nanoparticles over core–shell and bare nanoparticles. Such significant enhancement in the force maxima can have potential application in constant force measurements, for example, to determine the transition path dynamics of protein molecules using constant force measurement.^12,13^ Core–shell and core–shell–shell type of particles can be potential candidates to enhance the temporal and spatial resolution in such experiments. These results can be evident from the corresponding potential curves as well, as shown in Fig. 1. As we mentioned earlier, the trap's stability is analyzed from the *escape potential*,^7^ marked by a double-sided arrow with corresponding colors. It can be seen that the absolute depth of the potential well increases drastically, whereas escape potential increases slowly. The escape potential is ∼8 *k*_B_*T*, ∼9 *k*_B_*T*, and ∼11 *k*_B_*T* for polystyrene, ZnS–polystyrene, and ZnS–CdS–polystyrene nanoparticles, respectively. Although the change is small, it was shown that even such a small change in escape potential could lead to a significant change in the confinement time of the trapped particle.^28^ The noticeable point here is that the nanoparticles of same size can be trapped more efficiently if we use multiple layers instead of bare nanoparticles under similar trapping conditions.

**Fig. 2:**
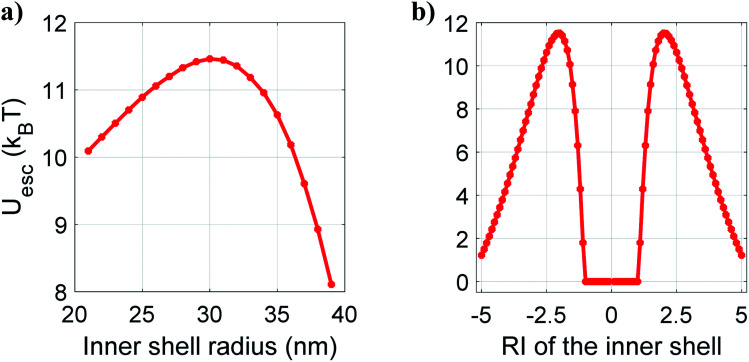
Variation of escape potential with (a) inner shell radius and (b) RI of the inner shell for core–shell–shell type nanoparticles under CW excitation at 100 mW average power.

Based on these results, it appears quite interesting to find the appropriate thickness and nature of the material of the inner shell that corresponds to the most stable trap. In order to estimate that, we now examine the variation of the escape potential with thickness and RI of the inner shell by fixing the overall nanoparticle size as 40 nm and the core size as 20 nm (considering polystyrene as the outer layer and ZnS as the core). Fig. 2a shows how escape potential varies against the variation of thickness of the inner shell (while keeping the inner shell material as CdS, *i.e.* considering ZnS–CdS–polystyrene nanoparticles) from 1 to 19 nm (*i.e.* varying *a*_s1_ from 21 nm to 39 nm while adjusting the thickness of the outer shell to keep the overall particle radius 40 nm) under CW excitation. The trapping efficiency varied with the thickness variation of the inner shell, and maximum efficiency is observed for 20–30–40 nm (*a*_c_–*a*_s1_–*a*_s2_) ZnS–CdS–polystyrene nanoparticle. Fig. 2b shows the plot of escape potential against the variation of RI of the inner shell for 20–35–40 nm ZnS–inner shell–polystyrene nanoparticle. With increasing RI, the escape potential increases reach to a maximum, then decreases. While increasing/decreasing the RI of the inner shell, at very high positive/negative RI the scattering force dominates over gradient force, results in destabilizing the trap, and eventually potential become unbound. The value of the RI of the inner shell is ∼±2, for which the trap is most stable. Of course, this optimal RI would be different for the different materials' compositions, the thickness of layers, and the nanoparticle's overall size. A crucial finding is that similar behavior is observed for the metamaterials (having negative refractive indices). Thus, different types of materials can be used according to the experimental requirement of longer trapping time.

Under pulsed excitation, the OKE is incorporated using eqn (5b); therefore, the nanoparticles' polarizability is inherently dependent on the (average) power of the trapping laser beam. For understanding the contribution of nonlinearity in polarizability, the polarizability can be rewritten in the form of linear and nonlinear terms. Eqn (4a) can be written as including the optical nonlinearity:^9^
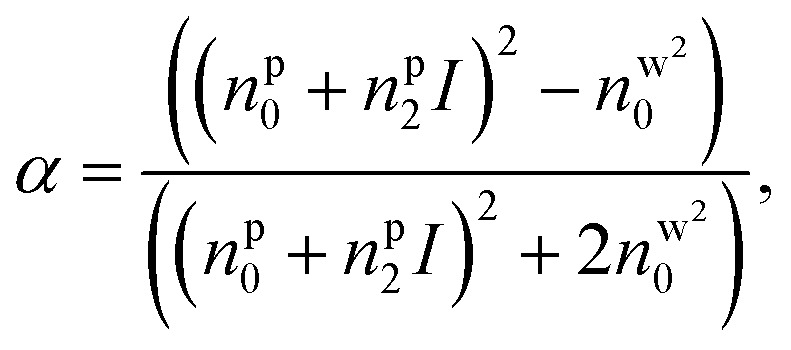

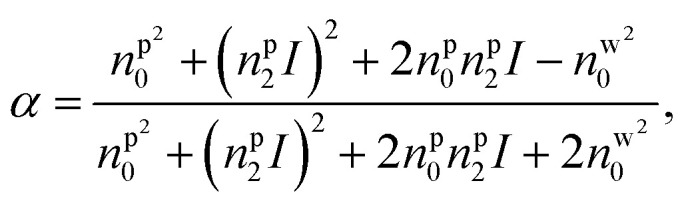
neglecting higher order terms (*n*^p^_2_*I*)^2^, and define 
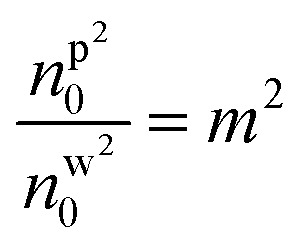

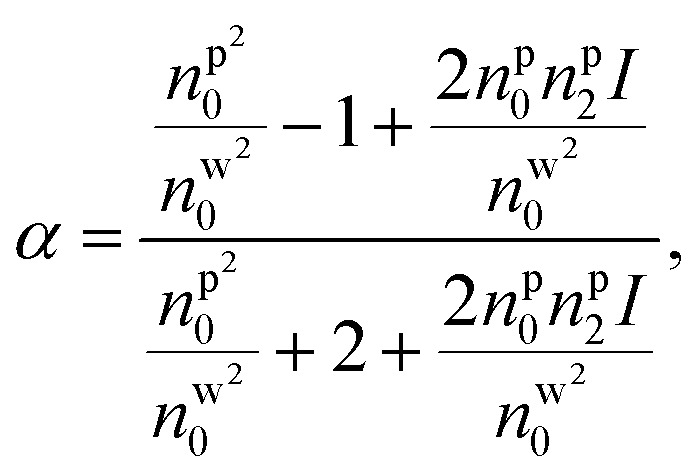

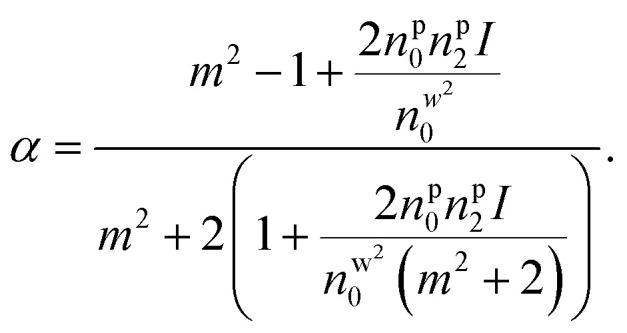


After Taylor expansion,8
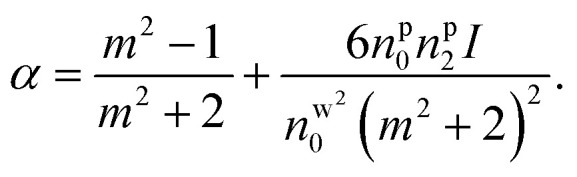


Here, the first term is the linear term of polarizability and the second term is the contribution of nonlinearity. The sign of second term gives a change in effective polarizability for the metamaterials and that gives a significant difference to the trapping efficiency. However, in case of metamaterials, the magnetic dipole effects are ignored due to the inherent limitations of the dipole approximation. These can be studied using more rigorous theories like generalized Lorenz-Mie theory or exact Mie theory.^10^

**Fig. 3:**
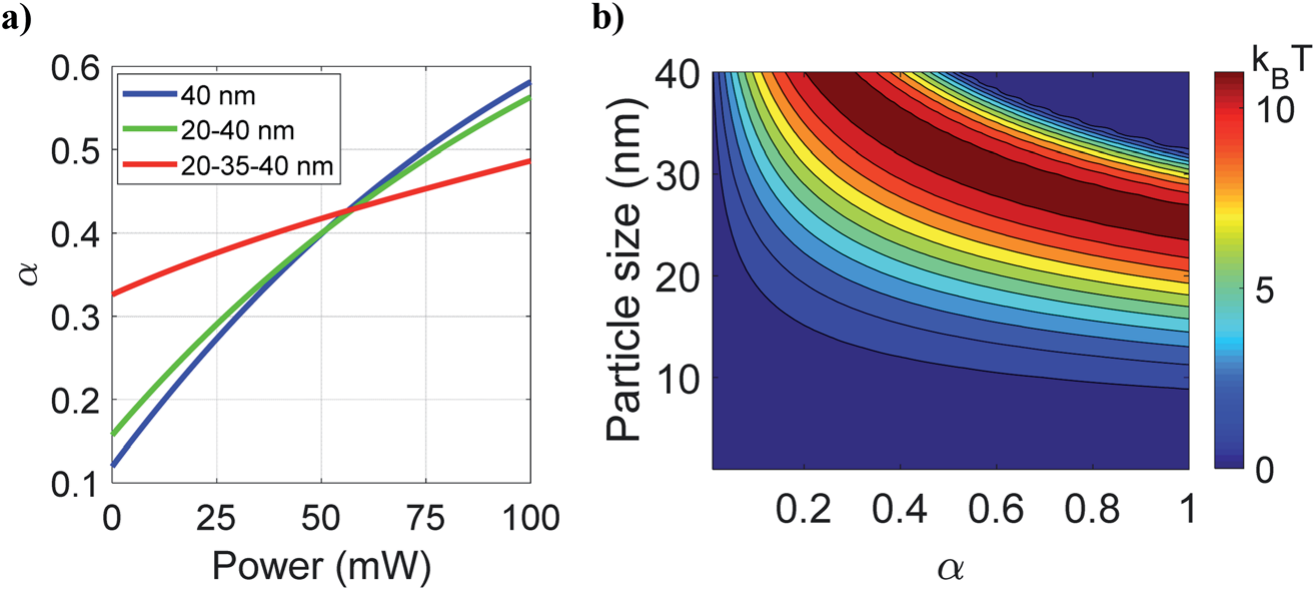
Variation of (a) polarizability per unit volume against average power for all three types of nanoparticles at a fixed 40 nm overall size under pulsed excitation. (b) Variation of escape potential with polarizability per unit volume and overall particle size at 100 mW average power. Color: blue/green/red curve represents the polystyrene/ZnS–polystyrene/ZnS–CdS–polystyrene nanoparticles, respectively.

Fig. 3a shows the variation of total effective *α* against average power for different types of nanoparticles. It can be observed that *α* increases with average power, but increment is more rapid for bare nanoparticles than the layered nanoparticles. This is because, in different types of nanoparticles, the radius of the polystyrene layer is 40 nm, 20 nm, and 5 nm in the bare, core–shell, and core–shell–shell type nanoparticles; polystyrene has the highest nonlinear RI among the materials chosen (polystyrene, ZnS and CdS). Therefore, the contribution of nonlinear RI in the total polarizability of hybrid nanoparticles is much dependent on the thickness of the outermost layer. The noticeable point is that at low average power, *α* (*i.e.* polarizability per unit volume) is significantly higher for core–shell–shell nanoparticles as compared to bare and core–shell type nanoparticles. However, increasing power reverses the case, and at high average power, bare nanoparticles show the dominancy of *α* over hybrid nanoparticles. Since forces are directly proportional to *α*, enhancement in *α* results in the enhancement in forces, which implies that at low average power (up to ∼60 mW) core–shell–shell gives better trapping efficiency than core–shell and bare nanoparticles. The total polarizability depends on the average power and the overall particle size and consequently the escape potential. Fig. 3b shows the variation of escape potential for different overall particle sizes as well as for varying *α*. The contour lines represent the same escape potential for different combinations of particle-size and *α*. For a given *α*, the escape potential increases up to a certain particle-size and then falls off; for example, for *α* ≈ 1, the maximum escape potential is for particle size of 25 nm. The maxima in escape potential shifts toward a lower value of *α* with higher value of particle size. This is because the escape potential decreases with increasing particle size since scattering force increases more rapidly than the gradient force. Therefore, for a given particle-size, trapping efficiency can be tuned by varying the material as well as thickness of the inner shell (which, in effect, changes *α*). This should not be compared with the fact that larger micro-particles are much easier to stably trap than smaller nano-particles since our results are strictly valid below a particle size of 80 nm (to validate the dipole approximation). For a fixed value of polarizability, larger particles can be more stably trapped in the nanoparticle regime but up to a limit of polarizability (∼0.3) as shown in Fig. 3b, beyond this limit of polarizability the efficiency (escape potential) first increases and then decreases with particle size and this optimal particle size decreases with polarizability. This is again due to the dominance of scattering force over gradient force as the scattering force increases with the square of particle size and polarizability.

**Fig. 4:**
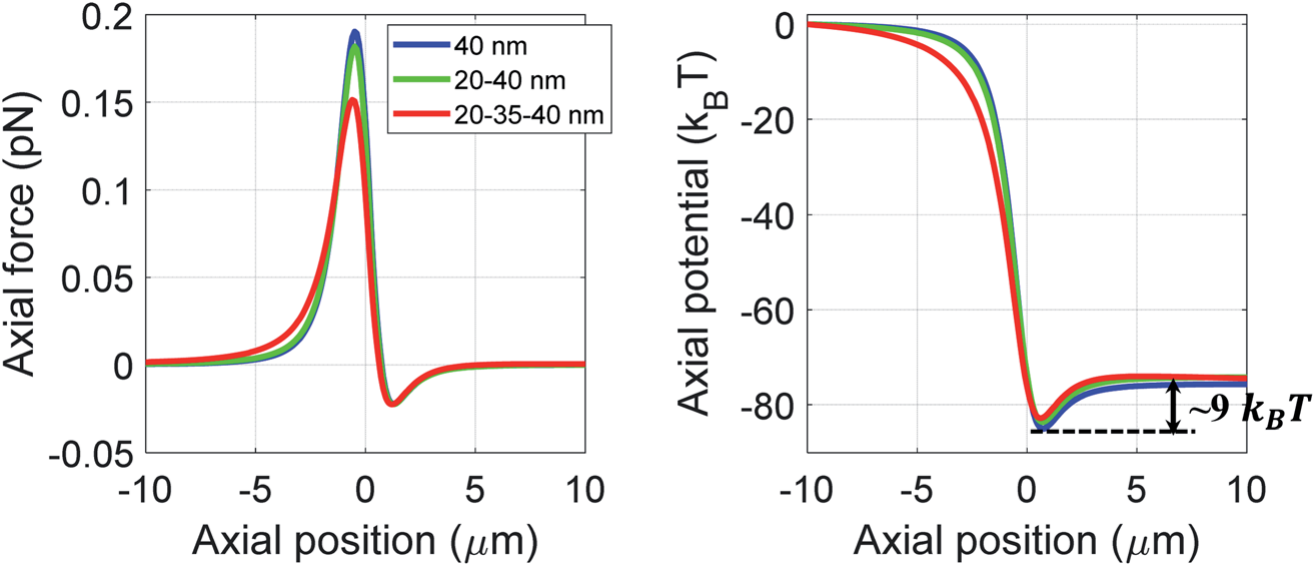
Trapping force or potential for polystyrene/ZnS–polystyrene/ZnS–CdS–polystyrene nanoparticles under pulsed excitation at 100 mW average power. Color: blue/green/red curve represents the polystyrene/ZnS–polystyrene/ZnS–CdS–polystyrene nanoparticles, respectively.

We now discuss the effect of optical nonlinearity under pulsed excitation for 20–35–40 nm (ZnS–CdS–polystyrene) nanoparticles. As shown in Fig. 4, the dragging force experienced by the particle is approximately twice than the CW excitation (see Fig. 1 for a comparison); this is also evident from the absolute depth of the potential well (∼55 *k*_B_*T versus* ∼85 *k*_B_*T*). However, a decrement in escape potential (and hence in confinement time) is observed as compared to CW excitation at 100 mW average power (∼11 *k*_B_*T versus* ∼9 *k*_B_*T*) because the nonlinear RI of polystyrene is higher than ZnS and CdS. This nonlinear term for polystyrene significantly contributes, rendering the total (linear plus nonlinear) RI of polystyrene much higher than ZnS and CdS which implies that light would scatter more from the outermost layer. Hence, scattering force dominates over gradient force which results in an unstable trap. From these results, it is evident that pulsed excitation can be either advantageous or disadvantageous over CW excitation and we can make our choice of nature of excitation judiciously. For example, if we want to use these particles for high force experiment for a short duration of trapping, we can take the advantage of impulsive dragging force under pulsed excitation. On the other hand, if we need the particles to be confined for a longer time, CW excitation is more suitable.

**Fig. 5:**
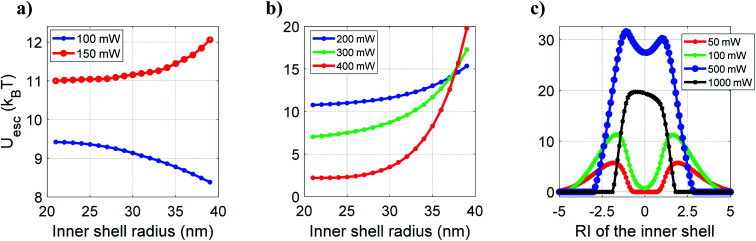
Plots of escape potential against (a and b) variation of the inner shell radius and (c) variation of RI of the inner shell at different average powers under pulsed excitation.

Now, to determine any dependence on the thickness of the inner shell (CdS layer) for which pulsed excitation may be more advantageous over CW excitation, we study the variation of escape potential with thickness of the inner shell which varies from 1 nm to 19 nm, as shown in Fig. 5a. Quite interestingly, at 100 mW average power, increasing thickness of CdS layer decreases the escape potential monotonically, however, at 150 mW average power, the case is reversed. Thus, one can readily identify the advantage of pulsed excitation over CW excitation and choose the type of excitation depending on the specific need (high-force experiments *versus* long confinement time experiments). This advantage is even more prominent for the CdS layer (*a*_s1_ greater than 30 nm), at high average power, as shown in Fig. 5b. Fig. 5c shows escape potential against RI of the inner shell (*n*^s1^_0_) at different average power under pulsed excitation. Please note that under pulsed excitation, the nonlinear RI of the inner shell is kept constant; hence, RI of the inner shell can be written as: *n*^s1^ = *n*^s1^_0_ + *n*^s1^_2_ × *I*_peak_(*r*, *z*) = *n*^s1^_0_ + 1.68 × 10^−18^ × *I*_peak_(*r*, *z*); here we consider the linear term (*n*^s1^_0)_ as a variable keeping the nonlinear term fixed. At 50 mW and 100 mW average powers, the trend of the curve is very similar to CW excitation (Fig. 2b), however, the value of *n*^s1^_0_ for which the trap is most stable shifts towards lower |*n*^s1^_0_|. Further increase in average power decreases the value of *n*^s1^_0_ corresponding to the most stable trap, and after a certain average power (∼500 mW), both the peaks merge into one peak. The nonlinear part of the RI of the inner shell (1.68 × 10^−18^ × *I*_peak_) increases with power, and the values are 0.0205, 0.0411, 0.2058, and 0.4116 at 50, 100, 500, and 1000 mW at average power, respectively. The effective *α* increases with the increasing contribution of nonlinear RIs of different layers with power; therefore, the optimal value of *α* corresponding to the most stable trap requires a less contribution from linear RI due to which the peaks shifts towards the lower value of |*n*^s1^_0_| with power and eventually decreases close to zero (marked by merging of peaks), as shown in Fig. 5c. Note that under CW excitation the curve of escape potential is symmetric (Fig. 2b) with *n*^s1^_0_ for both positive and negative values while it is asymmetric under pulsed excitation (Fig. 5c) which is due to the addition of positive nonlinear RI with positive or negative linear RI. From this, it is evident that by taking advantage of OKE, we can trap those particles that have RI very less than the surrounding medium; such particles cannot be trapped using CW excitation under similar conditions. In contrast, high RI (both linear and nonlinear) particles cannot be trapped under pulsed excitation at high average power because scattering force dominant over the gradient force. However, under CW excitation, a few high RI particles can be trapped with better trapping efficiencies under similar conditions.^9^

**Fig. 6:**
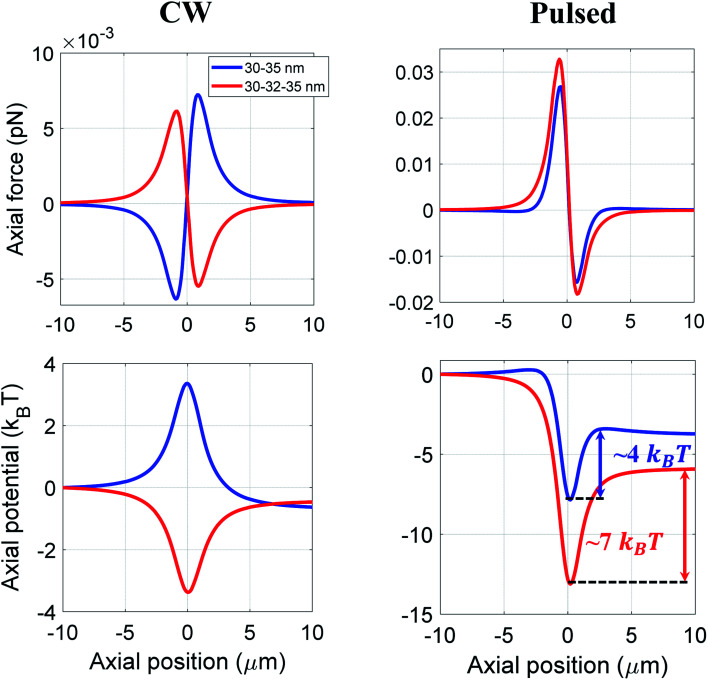
Trapping force and potential for hollow core–shell and hollow core–shell–shell type nanoparticles at 100 mW average power under CW and pulsed excitation.

Further, we examine the trapping of hollow core–shell–shell type nanoparticles by fixing the nanoparticle's overall size as 35 nm, considering many practical applications of hollow nanoparticles, for example in targeted drug delivery.^29^ Under CW excitation, 30–35 nm hollow-polystyrene nanoparticle experiences a repulsive force, as shown in Fig. 6. However, the presence of 2 nm CdS layer in-between the hollow-polystyrene nanoparticle allows the particle to experience an attractive force under similar excitation conditions. As mentioned earlier, the reversal nature of force (from repulsive to attractive) was shown for hollow-core nanoparticles upon changing the excitation from CW to pulsed (owing to the effect of OKE).^14–16^ In contrast, for a hollow core–shell–shell type nanoparticles, even under CW excitation such a reversal in the nature of the force is observed; note that similar reversal under CW excitation was also observed for hollow-core type nanoparticles by tuning the thickness of the material.^16^ Under pulsed excitation, owing to the OKE, both hollow-polystyrene and hollow-CdS–polystyrene nanoparticles experience an attractive force. Also, an enhancement in force is observed which is evident from the potential curves as well. Thus, it simultaneously offers both high force and long confinement time.

## Conclusion

In summary, the results presented in this article show that both CW and pulsed excitations have their own advantages and disadvantages, depending on the material properties, particle-size and excitation parameters (average power, *etc.*) and nature of excitation (pulsed *versus* CW). An appropriate choice of these parameters may enhances the ponderomotive optical force or enhance the duration of trapping and the effects are manifested to different extent depending on the type of particles (bare *versus* multilayered type). The noticeable point here is that the high index particles (after a limit of RI) do not always give better trapping efficiency because of high scattering force as discussed in terms of polarizability (Fig. 3b), so we have to choose the particle size and effective polarizability judiciously which can be obtained by varying the thickness of layers of different materials or/and through the effect of nonlinearity under pulsed excitation. These results are promised to break new grounds through controlled nanoscale optical manipulation.

As a final note, here we have used paraxial approximation in zeroth order Gaussian beam under high NA (1.4) tight focusing condition to study the qualitative comparison of results and in the case of metamaterials, the magnetic dipole effect is ignored due to the limitation of dipole approximation. However, more rigorous theories like generalized Lorenz–Mie theory or exact Mie theory or Maxwell stress tensor theory is needed for the exact calculation of quantitative results.

## Author contributions

S. Y. and A. D. contributed equally in this work.

## Conflicts of interest

The authors declare no competing interest.

## Supplementary Material
